# "Rod" or "Cane?"

**Published:** 1889-02-02

**Authors:** 


### The Hospital

A Weekly Institutional Journal of

**Science, Medicine, Nursing, and Philanthropy.**

*For Hospitals, Asylums, Medical Practitioners, Students, Nurses, Families, Parishes, Congregations, and the Charitable Public.*

Vol. V. No. 123.] [February 2, 1889.

### "Rod" or "Cane"?

There is no doubt that the youthful human being is a tough nut to crack. All men and women who have had practical experience in teaching, from the mistress of a dame's school to that most sorely tried of all grown-up men, the "army coach," will agree that labour, endurance, and disappointment are their daily and life-long companions. Even parents, whose love for their children is a constant source of patience, intelligence, and hope, are sometimes tempted to wish they had never known by personal experience the burden of family life. But the human race cannot get rid of responsibilty for its own successors. Here they are, always with us—in the home, in the Board school, in the private school, at Eton or Winchester, at Oxford or Edinburgh, at Sandhurst, or at Powis Square. They must be dealt with; and they must be dealt with as they are. If we make up our minds that we will not perform the labour, and endure the harassing worries which are inevitable parts of the care of childhood and youth, the only rational alternative is to shoot or drown all children as soon as they are born.

It is as plain as the sun at noonday that the training of children is one of the most difficult duties which Providence has assigned to mankind. The teaching of a young horse, which is meant for plough, or carriage, or saddle, is as nothing to it; the education of a pointer, a retriever, or a collie is child's-play compared with it. But then, the mind, the capability, the destiny of a horse or a dog—what are they compared with the mind, the capability, the destiny of a child? It never can be an easy task to train the young. Rough-and-ready methods are as impracticable as a ploughman at a painter's easel, or a mixer of mortar with the pencil of a Christopher Wren in his hand. No man or woman should be employed in any teaching capacity who is not possessed of teaching capability, and, above all, of a teaching temper and disposition. The cane cannot teach; the rod cannot teach; solitary confinement and deprivation of bread and butter—these can never be substituted for the just, intelligent, patient, and hopeful human teacher. Too many men and women who draw the wages of teachers from schools and colleges and universities are fitter for almost any other calling than for that greatest, most difficult, and most honourable of all occupations, the instructing and building up of the character of the young.

If we could place in every school for ordinary children, and in every reformatory for youthful delinquents, a teacher, or a set of teachers, fitted by mind and temper for the particular situation, the question of mere method would become a matter of very little moment. But that is just what never has been done, and what never can be done—and it is exactly there that difficulties begin. Men and women of the approved stamp cannot be found; and because men are not to be had the question of method assumes an importance which is altogether disproportionate, though inevitable. Teachers and their employers may be roughly divided into three classes—moral suasionists, physical compulsionists, and "all-round men"—that is, those who use any and every method which the circumstances of the case seem to demand. A recent controversy in the *Times* shows some of the exponents of those methods at their best and at their worst.

Now physiology has, and ought to have, a great deal to say on the subject of the education of the young, and particularly on that side of the question which deals with punishment. It is this latter aspect of the case that has been so keenly debated within the last few days. Can children be educated properly without punishment, and above all, children who are criminals, or who are the children of coarse, brutal, or criminal parents? Possibly here and there one such child may be amenable to the methods of moral suasion; but it is equally certain that a great many children are entirely beyond such methods, and will defy the teacher to his face who has neither the power nor the will to punish. Can a state be carried on without penal laws? Can an ordinary family be ruled and kept in order without occasional resort to severity? Let the objector remember this, that in childhood impulse and self-will are at the strongest, whilst reason, calculation, and foresight are at the weakest. If it be impossible to do without various and adequate punishments for grown up persons and persons in middle life, whose reason and foresight are fully developed, how much more impossible must it be to dispense with adequate punishments for the children of those very people, who are still at the period when self-will is in full play, and reason hardly operates at all? It was said in the earlier part of this article that neither the cane nor the rod could teach, and that is true; but the competent teacher can teach by means both of the rod and the cane.

There was a time when punishments of all kinds were unduly severe, and when children especially were treated with a cruelty which roused indignation to a white heat. The pendulum is now swinging, or has already swung, to the opposite extreme, and there are outcries heard on all hands that there ought to be practically no punishment at all. For to many children, the mild reproof, the standing out, the shutting up for a few hours in a solitary but well-lighted cell it would be absurd to call punishments. Doubtless there are sensitive boys and girls for whom these methods are sufficient; and if the teacher is fit for his post at all, he knows these sensitive ones, and treats them as their temperament requires.

But what about the majority, or, at any rate, a very formidable minority of children and youthful delinquents? Are those to be allowed to bid defiance to authority, and to go to ruin, lest one or more of the sensitive few should run the risk of occasionally severe or unjust treatment at incompetent hands? Punishment, and adequate punishment, is an essential part of the discipline of the whole of life. Nature punishes offenders, and with remarkable severity; law punishes; moralists punish, and some of them, because they cannot find evidence of sufficient punishment in this world, boldly, and not irrationally, carry their doctrines forward into the next. Parents punish; and those who are best endowed, morally and intellectually, most clearly see the necessity for adequate disciplinary punishment, and most sternly insist upon its being carried into effect.

With regard to the question whether the birch or the cane should be used, it is not worth a consideration except as a matter of detail. Both of them should of course be permitted, and the character of the subject and the special occasion should determine whether of the two it shall be. Punishment, we insist, must follow offence, and it must be of such a nature and severity as to convince the offender of the gravity of the ill he has done. There are many boys and girls who have not sufficient intelligence to thoroughly understand the baseness of a lie, or the wickedness of theft. For such as these sharp physical pain is absolutely necessary as a stimulus to the intellect. The cry for excessive mildness may be goodnatured, but it is of the very essence of imbecility. It is contrary to both nature and reason, and can have no other result but the ruin of those on whose behalf it is made. So far as the health aspect of the question is concerned, a good wholesome "skelping," as the author of "Rab and his Friends" called it, is often decidedly beneficial to a youngster. There are several parts of the anatomy where sharp, biting strokes may be freely and promptly given, not only without injury but with a certain amount of nerve stimulus, which is physiologically advantageous. It is very easy to make regulations by which severe punishment is entrusted to none but cool heads and competent hands. With such precautions, the public will do well to take little heed of sensational newspaper reports and exaggerated statements by interested friends. In all places where children and young people are congregated, whether in reformatories, board schools, or even higher class institutions, there will always be a large proportion whose mental and moral integument is decidedly thick, and whose response to strokes of a mental and moral character will be of the slowest and most limited character. In such cases the physical integument is generally supplied with nerves of a much more sensitive kind. To that integument, therefore, the appeal should be made with such a degree of promptitude, energy, and frequency as the circumstances of the case may demand.

